# Gut-Derived Uremic Toxins as a Risk Factor for Vascular Damage in Patients with Chronic Kidney Disease

**DOI:** 10.3390/ijms27083487

**Published:** 2026-04-13

**Authors:** María Carmen Ruiz Fuentes, Mahsa Rashki, Noelia Risquez Chica, Elena Clavero García, Elisa B. Pereira Pérez, María José Espigares Huete, Rosemary Wangensteen

**Affiliations:** 1 Nephrology Department, Hospital Universitario Virgen de las Nieves, 18012 Granada, Spain; 2 Instituto de Investigación Biosanitaria ibs.GRANADA, 18012 Granada, Spain; 3 Department of Medicine, University of Granada, 18071 Granada, Spain; 4 Area of Physiology, Department of Health Sciences, University of Jaen, 23071 Jaen, Spainrwangens@ujaen.es (R.W.)

**Keywords:** gut-derived uremic toxins, chronic kidney disease, dialysis, kidney transplantation, carotid intima media thickness, cardiovascular disease

## Abstract

Patients with chronic kidney disease (CKD) have a markedly increased cardiovascular risk that is not fully explained by traditional risk factors. Gut-derived uremic toxins, indoxyl sulfate (IS), indole-3-acetic acid (IAA), and p-cresyl sulfate (pCS), are poorly cleared by dialysis and may contribute to vascular damage. This cross-sectional observational study included 70 patients with CKD under different clinical conditions (pre-dialysis, peritoneal dialysis, hemodialysis, and kidney transplantation) and 17 healthy controls. Serum levels of IS, IAA, pCS and Klotho were measured, and vascular damage was assessed by carotid intima–media thickness (IMT) using ultrasound. CKD patients showed higher concentrations of IS, IAA, and pCS compared with controls, with the highest levels observed in hemodialysis patients. Peritoneal dialysis was associated with elevated IS and pCS, whereas in kidney transplantation, IS and IAA levels did not differ significantly from controls, and pCS remained elevated. Carotid IMT was higher in patients with diabetes and those undergoing hemodialysis. IAA correlated significantly with left/mean IMT, and mean IMT was the only parameter associated with previous cardiovascular events. These findings suggest that gut-derived uremic toxins, particularly IAA, might be associated with subclinical vascular damage in advanced CKD, although larger studies are needed to confirm these associations.

## 1. Introduction

Patients with chronic kidney disease (CKD) have a higher incidence of cardiovascular complications than the general population, a phenomenon that cannot be explained solely by the high prevalence of traditional cardiovascular risk factors [1]. Protein-bound uremic toxins account for approximately 25% of all currently identified uremic toxins. Many of these toxins are of intestinal origin and result from the metabolism of aromatic amino acids by the gut microbiota [2,3].

Indoxyl sulfate (IS) is a final product of tryptophan metabolism by the intestinal microbiota; it undergoes an initial transformation in the colon followed by a second metabolic step leading to the formation of indoxyl sulfate [2,4]. Another tryptophan-derived compound is indole-3-acetic acid (IAA), which is directly released into the colon [5]. p-Cresyl sulfate (pCS) is the final product of tyrosine and phenylalanine metabolism by the gut microbiota [3,4].

These uremic toxins are highly protein-bound, accumulate as the glomerular filtration rate declines in CKD, and contribute to the progression of kidney disease and the development of cardiovascular complications through multiple inflammatory and oxidative stress pathways [2,4,6]. An inverse correlation between the concentrations of these toxins and serum Klotho levels has been described in this population [2,3]. Although the association of these toxins with vascular damage and calcification has been well documented, some controversy remains regarding their relationship with cardiac mortality, first cardiovascular events, and sudden cardiac death [3].

Measurement of carotid intima–media thickness (IMT) provides an accessible assessment of vascular status and serves as a marker of subclinical atherosclerosis and cardiovascular risk [7,8,9]. Several studies have independently demonstrated associations between circulating levels of indoxyl sulfate and p-cresyl sulfate and carotid IMT in patients with CKD [10].

Once patients reach stage 5 of the KDIGO classification of chronic kidney disease, initiation of renal replacement therapy becomes necessary. Uremic toxins of intestinal origin, due to their high degree of protein binding, exhibit limited clearance with dialysis techniques, resulting in higher circulating concentrations in patients receiving dialysis, intermediate levels in pre-dialysis patients, and lower levels in kidney transplant recipients [3,5].

Our hypothesis is that uremic toxins of intestinal origin, which are increased in patients with chronic kidney disease, exhibit different concentrations depending on whether patients are in the pre-dialysis phase, dialysis, or receiving renal replacement therapy and that these concentrations correlate with carotid intima–media thickness and with the prevalence of cardiovascular events in this population.

The aim of the study was to evaluate serum concentrations of specific biomarkers, Klotho, and uremic toxins of intestinal origin (indoxyl sulfate, indole-3-acetic acid, and p-cresyl sulfate) in patients with chronic kidney disease (CKD) across different clinical settings (pre-dialysis, peritoneal dialysis, hemodialysis, and kidney transplantation) to compare these findings with a control group of healthy individuals and to assess the relationship between these toxins and parameters of cardiovascular disease.

**Figure 1:**
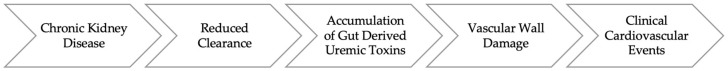
Key mechanisms linking CKD with cardiovascular risk.

To facilitate the interpretation of the pathophysiological framework underlying this study, Figure 1 summarizes the key mechanisms linking chronic kidney disease with vascular injury and cardiovascular risk.

## 2. Results

### 2.1. Demographic Data

A total of 70 cases were studied, of which 14.28% were in a pre-dialysis situation, 17.14% were undergoing hemodialysis, 34.29% peritoneal dialysis, and 34.29% were kidney transplant recipients. The control group consisted of 17 individuals without a known pathology.

The descriptive data are shown in the next tables: Table 1 for qualitative data and Table 2 for quantitative data.

In the control group, 23.5% were men and 76.5% women. No one had diabetes, hypertension, or any cardiovascular event.

### 2.2. Case–Control Analysis

#### 2.2.1. Complete Group Case–Control Analysis

Next, we compared all patients with chronic kidney disease, regardless of clinical setting, with the control group. In this analysis, we observed the expected significant differences in routinely assessed biochemical parameters, with higher values in the cases than in healthy controls. Notably, urea levels were significantly increased in cases (98.30 ± 40.70 vs. 33.76 ± 9.64 mg/dL, *p* < 0.00001), as were calcium (9.14 ± 0.79 vs. 8.76 ± 0.32 mg/dL, *p* = 0.003), phosphorus (*p* = 0.0018), potassium (4.64 ± 0.72 vs. 4.12 ± 0.29 mEq/L, *p* = 0.001), albuminuria (36 (184.35) vs. 0.5 (3.6) mg/dL, *p* = 0.0003), and parathyroid hormone levels (225.25 (238.6) vs. 72.25 (34.05) pg/mL, *p* < 0.0003). Regarding the estimated glomerular filtration rate, the control group showed significantly higher CKD-EPI values compared with all cases (98.47 (13.83) vs. 13.12 (38.98) mL/min/1.73 m^2^), as well as compared with patients in pre-dialysis (17.97 (5.96) mL/min/1.73 m^2^) and those with kidney transplantation (51.94 (22.36) mL/min/1.73 m^2^; *p* < 0.0001) (Table 2).

Gut-derived uremic toxins were assessed, and circulating concentrations of indoxyl sulfate, indole-3-acetic acid, and p-cresyl sulfate were significantly higher in the CKD group compared with controls (Table 3).

After stratifying cases by clinical setting, the following comparative results were observed between each group and the control group.

#### 2.2.2. Peritoneal Dialysis Group and Controls

No significant differences were observed in Klotho concentrations.

**Figure 2:**
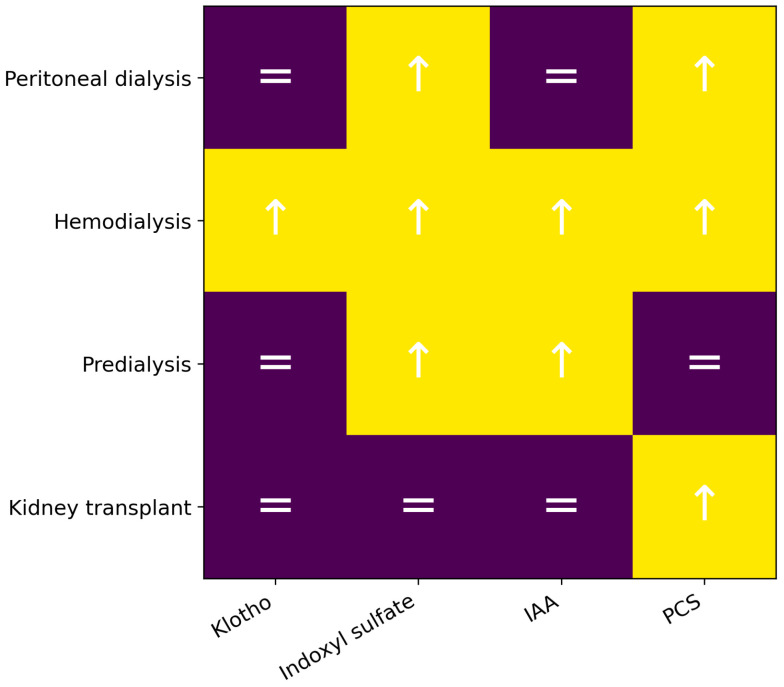
Heatmap of biomarker differences between cases and controls according to clinical setting. Arrows indicate significantly higher concentrations in the case group, whereas equal signs indicate no statistically significant differences.

IS concentrations were significantly higher in patients undergoing peritoneal dialysis compared with controls (*p* < 0.0001), as were pCS levels (*p* = 0.0380); no significant differences were observed for indole-3-acetic acid. These results are shown in Table 4 and Figure 2.

#### 2.2.3. Hemodialysis Group and Controls

Klotho levels were higher in hemodialysis patients (*p* = 0.0321). Indoxyl sulfate exhibited the same behavior as in peritoneal dialysis (*p* < 0.00001). IAA was significantly higher in cases (*p* = 0.0030), as was pCS (*p* = 0.0006) (Table 5 and Figure 2).

#### 2.2.4. Pre-Dialysis Group and Controls

No significant differences were found in Klotho concentrations. Higher IS concentrations were observed in pre-dialysis patients (*p* = 0.0088), as well as higher IAA concentrations (*p* = 0.0103), whereas pCS did not show a significant difference (*p* = 0.1221) (Table 6 and Figure 2).

#### 2.2.5. Kidney Transplantation and Controls

In this case, no significant differences were observed in Klotho levels (*p* = 0.2617). Regarding uremic toxins, no significant differences were found for IS (*p* = 0.99) or IAA (*p* = 0.36); however, pCS showed a significant difference (*p* = 0.0002), with higher concentrations in the case group (Table 7 and Figure 2).

### 2.3. Chronic Kidney Disease Patient Analysis

#### 2.3.1. Bivariant Analysis

Firstly, qualitative and quantitative variables were analyzed in the entire cohort, regardless of treatment group. A bivariate analysis was performed to compare differences in Klotho and uremic toxins according to the different categorical variables; no statistically significant differences were observed in the central tendency measures of uremic toxins across categorical variables. Differences according to treatment group were observed and will be analyzed later.

**Figure 3:**
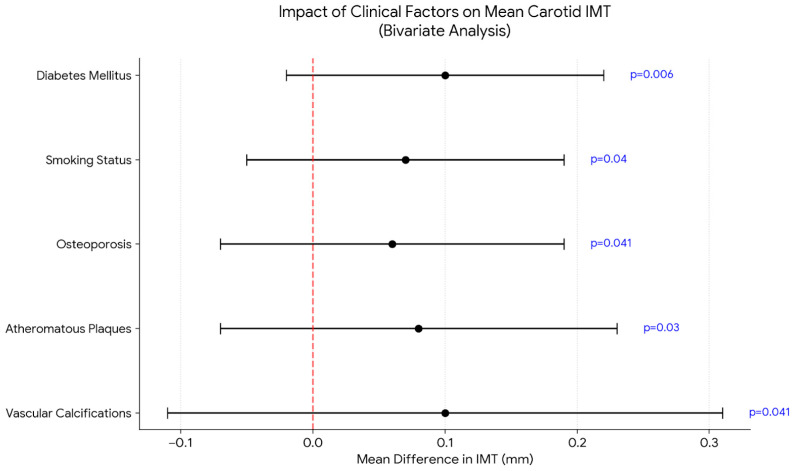
Forest plot of mean carotid intima–media thickness (IMT) differences. The figure illustrates the mean difference (mm) in carotid IMT according to various clinical determinants. Points represent the mean difference between groups (factor present vs. absent), while horizontal bars indicate the standard deviation. All factors shown were significantly associated with increased IMT (*p* < 0.05). The red dashed line represents the null hypothesis (no difference).

In the bivariate analysis involving carotid intima–media thickness (IMT), the following findings were observed: left carotid IMT was higher in patients with diabetes (*p* = 0.0421) and in those undergoing hemodialysis (*p* = 0.001). Right carotid and mean IMT were higher in patients with diabetes (*p* = 0.0183 and *p* = 0.006), smokers (*p* = 0.0242 and *p* = 0.04), osteoporosis (*p* = 0.041), atheromatous plaques (*p* = 0.0147 and *p* = 0.03), and vascular calcifications (*p* = 0.0001 and *p* = 0.041), as shown in Figure 3 and Table A1.

#### 2.3.2. Correlation Analysis

**Figure 4:**
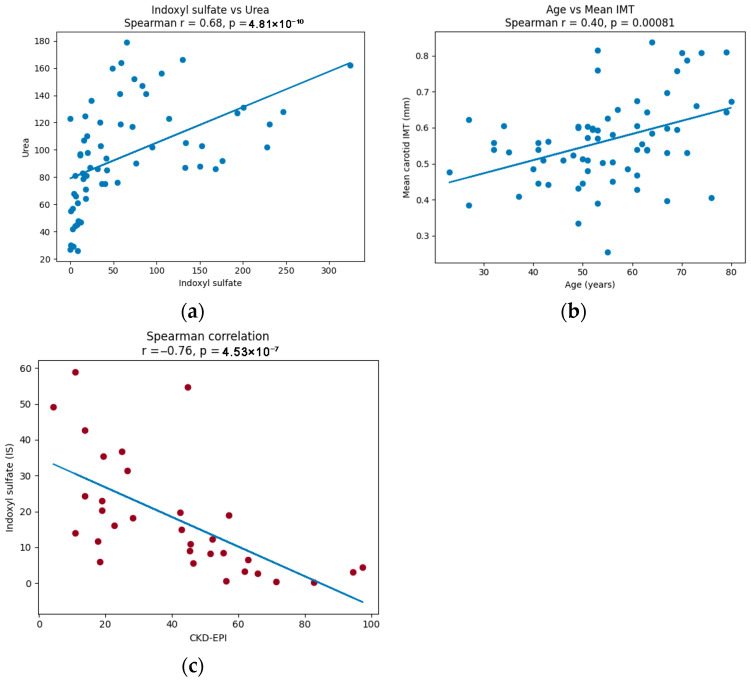
Correlation analyses: (**a**) IS vs. urea; (**b**) age vs. mean IMT; and (**c**) CKD-EPI vs. IS in non-dialysis patients.

The strongest and most expected positive correlation was observed between indoxyl sulfate and urea (*p* < 0.0001, r = 0.68), as shown in Figure 4a. Lower, although still significant, correlation coefficients were found with magnesium (*p* < 0.0001, r = 0.62), albuminuria (*p* < 0.0001, r = 0.55), phosphorus (*p* < 0.0001, r = 0.49), and parathyroid hormone (PTH) (*p* = 0.003, r = 0.36). An inverse correlation was observed with serum albumin (*p* = 0.007, r = −0.33) and calcium (*p* = 0.001, r = −0.50).

Right, left, and mean carotid intima–media thickness (IMT) showed a significant but weak correlation with age: right IMT: r = 0.28, *p* = 0.02; left IMT: r = 0.38, *p* = 0.002; and mean IMT: r = 0.40, *p* = 0.0008 (represented in Figure 4b). Right and mean IMT were also weakly correlated with body mass index (BMI) (*p* = 0.01, r = 0.31 and *p* = 0.04, and r = 0.25, respectively).

Regarding uremic toxins, indole-3-acetic acid (IAA) showed a weak but significant correlation with left IMT (r = 0.30, *p* = 0.016) and mean IMT (r = 0.31, *p* = 0.01).

When we only analyzed patients not receiving dialysis (pre-dialysis and kidney transplantation), we found a significant inverse correlation between indoxyl sulfate (IS) and the estimated glomerular filtration rate assessed by CKD-EPI (r = −0.78, *p* < 0.0001) (Figure 4c). CKD-EPI was also correlated with indole-3-acetic acid (IAA), while IS showed a direct correlation with IAA (Table 8).

No correlation was observed between uremic toxin concentrations and time on renal replacement therapy; however, left IMT showed a weak but significant correlation with time on renal replacement therapy (*p* = 0.033, r = 0.26).

#### 2.3.3. Uremic Toxins According to Clinical Setting Group

Indole-3-acetic acid (IAA) did not show significant differences across the different clinical settings of the patients included in our study; however, it approached statistical significance (*p* = 0.0738), with a trend toward higher concentrations in patients undergoing hemodialysis.

Indoxyl sulfate showed significant differences across clinical settings (*p* < 0.0001), with significantly higher concentrations in hemodialysis compared with pre-dialysis (*p* = 0.0014) and renal transplantation (*p* < 0.0001). No significant differences were observed between hemodialysis and peritoneal dialysis. In comparison with renal transplantation, patients on peritoneal dialysis also showed significantly higher indoxyl sulfate concentrations (*p* = 0.0001). pCS concentrations were also higher in patients undergoing hemodialysis. Details of the data are in Table A2.

#### 2.3.4. IMT According to Clinical Setting Group

**Figure 5:**
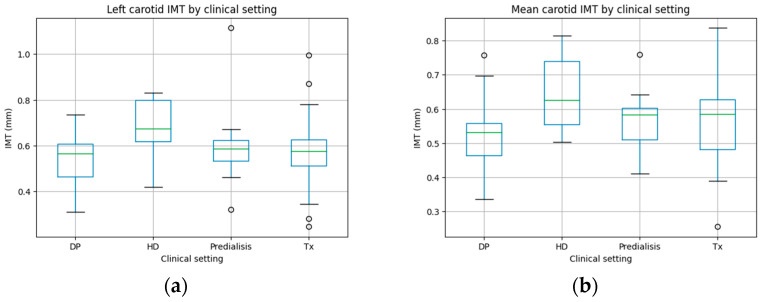
Carotid IMT differences according to clinical status group. (**a**) Left carotid IMT, (**b**) mean carotid IMT. DP: peritoneal dialysis; HD: hemodialysis; and Tx: transplantation.

Right carotid intima–media thickness (IMT) did not show significant differences according to pre-dialysis status, dialysis modality, or renal transplantation (*p* = 0.1499). Both left carotid IMT (*p* = 0.0480) and mean carotid IMT (*p* = 0.0420) were higher in patients undergoing hemodialysis, particularly when compared with peritoneal dialysis. These results are represented in Figure 5, and the data is in Table A3.

#### 2.3.5. Cardiovascular Events

**Figure 6:**
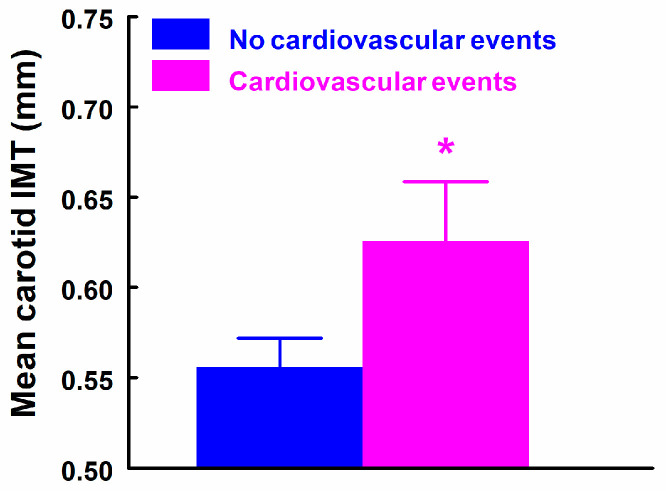
Mean carotid intima–media thickness according to cardiovascular events. Bars represent mean values, and error bars indicate standard error. Patients with cardiovascular events showed higher mean carotid IMT (*p* = 0.042). * *p* < 0.05 in patients with cardiovascular events (n = 8) vs. patients with no cardiovascular events (n = 59).

Given the small number of patients with individual cardiovascular events (stroke, arrhythmias, acute coronary syndrome, and peripheral ischemia), all cardiovascular events were grouped into a single composite variable to assess their association with uremic toxins and left and mean carotid intima–media thickness (IMT). Only mean IMT was significantly associated with cardiovascular events (*p* = 0.042), as shown in Figure 6.

Stratified analyses of qualitative variables by group were not feasible because of the small sample size within each group.

## 3. Discussion

The present study is consistent with classical publications showing that the concentration of gut-derived protein-bound uremic toxins is increased in patients with chronic kidney disease compared with healthy controls [2,3,11]. Moreover, among patients with CKD, these concentrations were higher in those receiving dialysis therapies—particularly hemodialysis—than in pre-dialysis patients and especially when compared with kidney transplant recipients [11]. In transplant recipients, only pCS levels differed compared with healthy controls. Carotid intima–media thickness (IMT), as a marker of subclinical vascular disease, was associated with age [12,13]. Differences according to carotid laterality were observed in its relationship with other cardiovascular risk factors in the presence of vascular lesions diagnosed by plain radiography and in the clinical status of chronic kidney disease. IMT only showed a weak correlation with indole-3-acetic acid (IAA). No association was found between cardiovascular events and uremic toxins, whereas an association was observed with mean IMT.

In addition to the elevated routine parameters characteristic of CKD, gut-derived protein-bound uremic toxins are increased in these patients [2,3]. Tryptophan-derived uremic toxins include indoxyl sulfate (IS) and indole-3-acetic acid (IAA). Indole is a metabolic product of tryptophan degradation by bacterial tryptophanase; it is absorbed into the portal circulation and, once in the liver, is hydroxylated to IS [2,3]. IAA is derived directly from tryptophan in the colon [5]. p-Cresyl sulfate (pCS) originates from p-cresol, a derivative of the intestinal bacterial metabolism of tyrosine and phenylalanine, which is absorbed and subsequently sulfated in the liver to produce pCS [2,3].

These gut-derived uremic toxins are elevated in CKD compared to healthy controls, with the exception of kidney transplant recipients [5,11]. In these patients, a progressive decrease—particularly in indoxyl sulfate (IS) and p-cresyl sulfate (pCS)—has been described, resulting in concentrations that do not differ significantly from healthy controls [11]. This reduction is attributed to improved renal function and various factors influencing the gut microbiota, such as the transplant procedure itself, immunosuppressive therapy, and antibiotic use [5,11]. Numerous experimental and clinical studies indicate that microbiota dysbiosis is closely linked to allogeneic transplantation and post-transplant complications [5].

Given that these uremic toxins are highly protein-bound, they are not effectively cleared by conventional dialysis techniques [2,3,4]. This explains why patients on hemodialysis exhibit the highest concentrations compared to those in the pre-dialysis or post-transplantation stages. Our study reflects these findings: only pCS levels were significantly elevated in kidney transplantation compared to controls, while tryptophan-derived toxins showed no significant differences. Most literature prioritizes the analysis of IS over indole-3-acetic acid (IAA) [5]. Although published studies comparing IS and pCS in kidney transplantation versus healthy controls report no significant differences post-transplantation, pCS levels frequently remain higher in the transplant group [11]. In our case, this discrepancy may be related to the sample size.

The inverse correlation between the increase in gut-derived uremic toxins and the decline in the glomerular filtration rate (GFR) is well-documented; this rise in concentration, in turn, promotes the progression of kidney disease [1,2,4]. Both IS and pCS are associated with nephrotoxic effects through several mechanisms, including the generation of reactive oxygen species (ROS), depletion of antioxidant systems, and the induction of inflammation—mediated by adhesion molecules such as ICAM-1—and fibrosis via the upregulation of NF-κB, TGF-β1, and PAI-1 [2,3,14]. Our study sample showed a significant inverse correlation between IS and IAA and the estimated glomerular filtration rate (CKD-EPI) in patients not receiving dialysis (pre-dialysis and kidney transplant recipients). We also identified a significant correlation between urea and IS in all patients.

Chronic kidney disease (CKD) is characterized by a high incidence of cardiovascular comorbidity, which cannot be explained solely by the traditional cardiovascular risk factors shared with the general population [6,12]. Elevated concentrations of gut-derived uremic toxins, IS and pCS, have been shown to influence endothelial inflammation by increasing ROS, decreasing nitric oxide (NO) levels, and upregulating MCP-1 and adhesion molecules, thereby enhancing leukocyte interaction with the vascular endothelium [15]. Furthermore, they contribute to vascular calcification by promoting the phenotypic transition of vascular smooth muscle cells into an osteogenic phenotype, while exerting pro-inflammatory and pro-fibrotic effects on cardiomyocytes and cardiac fibroblasts [2,3]. Collectively, these processes drive the progression of cardiovascular disease. Investigating analytical or ultrasonographic biomarkers to define cardiovascular risk in these patients will enable more personalized strategies for the prevention and management of these factors [9,12].

According to treatment modality in patients with CKD, previous studies consistently report that patients undergoing hemodialysis have higher concentrations of gut-derived uremic toxins—particularly IS and pCS—than those on peritoneal dialysis, with both groups showing higher concentrations than pre-dialysis patients [11]. In our population, IAA did not differ according to clinical status. IS did not show differences between dialysis modalities but was significantly higher in hemodialysis compared with pre-dialysis and kidney transplant recipients, whereas pCS concentrations were significantly higher in hemodialysis than in all other groups. These findings are consistent with the high protein-binding properties of these toxins, which result in reduced clearance with dialysis techniques. We did not find differences in Klotho concentrations among the different groups, unlike several published studies [2,3].

Increased common carotid artery IMT has been associated with the presence of cardiovascular risk factors [7,13]. Several studies have shown that elevated concentrations of gut-derived uremic toxins, particularly IS and pCS, are independently associated with increased IMT in patients with CKD, even after adjustment for traditional cardiovascular risk factors, suggesting a direct role of these toxins in vascular remodeling [16]. In general, an increased IMT measurement in either the right or left common carotid artery is considered clinically relevant; however, differences related to laterality have been described. Due to anatomical factors, IMT is more frequently increased in the left carotid artery, as its direct origin from the aortic arch results in greater shear stress. In contrast, increased IMT in the right carotid artery has been more closely associated with biochemical risk factors (such as diabetes mellitus and dyslipidemia), whereas left-sided IMT appears to be more strongly related to hemodynamic factors [8,10]. The specific differences in IMT laterality in CKD are not clearly established in the literature.

In our population, IMT was significantly higher in patients with diabetes mellitus and in those undergoing hemodialysis, while right-sided IMT and mean IMT were significantly associated with diabetes mellitus, smoking, osteoporosis, the presence of atherosclerotic plaques, and vascular calcifications. IMT correlated with age in our cohort; however, only left carotid IMT (and mean IMT) showed a significant, albeit weak, correlation with IAA. Compared with IS and pCS, IAA has received less attention in studies on uremic toxins and CKD. In a study including 120 patients across different CKD stages, higher IAA concentrations were associated with significantly increased mortality and cardiovascular events [17]. Although direct evidence linking IAA to carotid IMT is limited, experimental and clinical studies indicate that IAA promotes endothelial dysfunction via oxidative stress, inflammation, and activation of the aryl hydrocarbon receptor pathway, suggesting an association with vascular remodeling processes [15,17,18].

In patients with different stages of CKD, increased concentrations of gut-derived uremic toxins have been associated with vascular involvement and cardiovascular complications [2,4,16]; however, some studies have failed to confirm an association between these concentrations and cardiac events [3]. In our cohort of patients with CKD, the concentrations of uremic toxins measured were not associated with the prevalence of cardiovascular events, although an association was observed with mean IMT, which in turn correlated with IAA.

The main limitations of this study include its cross-sectional design and the relatively small sample size, which was further stratified into several treatment groups, thereby precluding the performance of multivariable analyses. There is a sex imbalance between the controls and cases that may introduce residual confounding; future studies with more balanced recruitment are needed to address sex-related differences. In addition, the modest sample size and the small number of patients in each clinical subgroup limit the statistical power of the analyses and increase the risk of both type I and type II errors. Consequently, the associations reported in this study should be interpreted as non-adjusted exploratory findings; therefore, caution is required when drawing conclusions. The large number of statistical tests increases the risk of false-positive findings, and multiple-comparison corrections were not applied due to the exploratory nature of the study. These factors may also have contributed to the weak strength of the significant correlations observed.

## 4. Materials and Methods

### 4.1. Study Design

An observational, cross-sectional study was conducted including 70 cases with chronic kidney disease in various clinical situations—pre-dialysis (n = 10), peritoneal dialysis (n = 24), hemodialysis (n = 12), and kidney transplantation (n = 24)—and 17 healthy controls. All patients were informed about the study and provided written informed consent.

### 4.2. Variables

In the control group, age, sex, and the absence of comorbidities such as diabetes mellitus, arterial hypertension, cerebrovascular disease, or chronic kidney disease were recorded.

In the case group, clinical variables obtained from the medical record were collected: sex, diabetes mellitus, smoking status, renal replacement therapy, presence of atheromatous plaques, history of stroke, ischemic heart disease, arteriovenous fistula thrombosis, presence of arrhythmias, peripheral artery disease, presence of vascular calcifications on plain radiography, osteoporosis, death, and use of different treatments (statins, oral iron, antibiotics, beta blockers, calcineurin inhibitors, mycophenolate mofetil, and corticosteroids). Quantitative variables included age, body mass index, systolic blood pressure, diastolic blood pressure, duration of renal replacement therapy, and carotid intima–media thickness.

### 4.3. Analysis

In both groups, the following parameters were measured according to the reference hospital protocol: glucose, urea, creatinine, hemoglobin, sodium, potassium, chloride, pH, bicarbonate, lactic acid, C-reactive protein, cholesterol, triglycerides, total proteins, albumin, albuminuria, proteinuria, calcium, phosphorus, parathyroid hormone, vitamin D, and magnesium, as well as baseline Klotho, and the gut-derived uremic toxins indoxyl sulfate, indole-3-acetic acid, and p-cresyl sulfate.

### 4.4. IMT Measure

Each patient underwent B-mode ultrasonography (Sonoline Antares, Siemens, Germany) using a 7.5 MHz linear transducer to examine the carotid artery. The left and right common carotid arteries, the carotid bifurcation, and the internal and external carotid arteries were scanned to assess IMT, defined as the distance between the first echogenic line separating the arterial wall from the lumen (intima–lumen interface) and the second echogenic line separating the media from the adventitia (media–adventitia interface).

### 4.5. Klotho and Uremic Toxins Determination

The determination of Klotho was performed with a commercial ELISA kit purchased from Boster Biological Technology (Pleasanton, CA, USA) using a standard curve from 0.156 to 10 ng/mL.

Indoxyl sulfate (IS), índole-3-acetic-acid (IAA), and p-cresyl sulfate (pCS) were measured using a Shimadzu HPLC system (Kyoto, Japan) with a fluorescence detector, using the method validated by Silva et al. [19]. A reversed-phase analytical column (Shim-pack VP-ODS, 150 × 2.0 mm i.d., Shimadzu) was employed for chromatographic separation at room temperature (20 ± 2 °C). The mobile phase was a mixture of acetonitrile-aqueous phosphate buffer (NaH_2_PO_4_, 20 mM) (30:70, *v*/*v*), and isocratic elution was implemented at a flow rate of 1 mL/min. Fluorescence detection was monitored at the emission wavelengths of 275 and 330 nm, respectively, with a gain set of ×1000. Total run time was 12 min, and the injection volume was 20 μL. Human plasma standards and samples containing uremic toxins (50 µL) were added to 150 µL of ethanol containing 0.8 µg/mL of the internal standard 4-ethylphenol (corresponding to 0.2 µg/mL in final extract) to precipitate proteins. After vortexing for 30 s, 50 mg of NaCl was added and mixed vigorously, promoting salting-out-assisted deproteinization. After 10 min, 350 μL of the aqueous component of mobile phase was further added. The extract was then vortexed and centrifuged at 18,000× *g* for 10 min at 4 °C. After centrifugation, the resulting supernatants were analyzed by HPLC. We used calibration curves of six concentration levels for each analyte: 0.5, 1, 2, 4, 8, and 10 µg/mL for IS; 0.2, 0.5, 1, 2, 3, and 4 µg/mL for IAA; and 10, 20, 30, 40, 50, and 60 µg/mL. All chemicals were acquired from Merck-Sigma-Aldrich (St. Louis, MO, USA).

### 4.6. Statistical Analysis

A descriptive analysis of all study variables was performed. Measures of central tendency and dispersion were calculated for numerical variables and absolute and relative frequencies for qualitative variables. Normality was assessed using the Shapiro–Wilk or Kolmogorov–Smirnov test depending on sample size in order to determine whether parametric or non-parametric tests would subsequently be applied.

To compare quantitative outcomes between two groups of patients, Student’s *t* test, Welch’s test, or the Mann–Whitney U test were used as appropriate. For comparisons involving more than two groups, ANOVA or the non-parametric Kruskal–Wallis test was applied. Pearson or Spearman correlation coefficients were estimated as required.

A *p* value < 0.05 was considered statistically significant. Statistical analyses were performed using STATA version 16.1.

Generative AI tools were only used to support the creation of graphical materials and reference organization. All underlying data and interpretations were produced and verified by the authors.

## 5. Conclusions

Gut-derived uremic toxins (IS, IAA, and pCS) are significantly elevated in patients with CKD. Hemodialysis is the modality associated with the highest concentrations of these toxins due to limited clearance resulting from high protein binding. Increased carotid IMT is associated with hemodialysis status and diabetes mellitus. Furthermore, IAA could emerge as a biomarker linked to IMT, suggesting its potential association with vascular parameters observed in these patients. In contrast, p-cresyl sulfate showed a paradoxical pattern, remaining elevated in kidney transplant recipients despite the recovery of renal function, underscoring the influence of other kidney-independent factors.

Studies with larger sample sizes are required to confirm the possible association between uremic toxins, ultrasonographic biomarkers, and cardiovascular events in patients with CKD, as well as to explore this relationship in greater depth through longitudinal designs, microbiome profiling, and mechanistic analyses of tryptophan-derived toxins. Such evidence would facilitate a more personalized approach to cardiovascular risk assessment and the implementation of targeted interventions in this patient population.

## Figures and Tables

**Table 1:** Qualitative demographic data of case group. AVF: arteriovenous fistula.

Variable	Women% (n)
Sex	42.9 (30)
	**Yes% (n)**
Diabetes	21.4 (15)
Smoker	31.4 (22)
Pre-dialysis	14.3 (10)
Hemodialysis	17.1 (12)
Peritoneal dialysis	34.3 (24)
Kidney transplantation	34.3 (24)
Atheromatous plaque	32.9 (23)
Stroke	2.9 (2)
Ischemic heart disease	1.4 (1)
Angor	2.9 (2)
AVF thrombosis	12.9 (9)
Arrythmia	5.7 (4)
Peripheric arteriopathy	4.3 (3)
Exitus	2.9 (2)
Antihypertension treatment	72.9 (51)
Vascular calcification	41.4 (29)
Osteoporosis	48.6 (34)

**Table 2:** Quantitative variables of case group and control group. * Mean ± standard deviation. ** Median (interquartile rank).

Variable	Case (n = 70)	Control (n = 17)	*p* Value
Age (years)	54.21 ± 13.71 * 53.50 (15.00) **	51.47 ± 9.96 54.00 (12.00)	
BMI (kg/m^2^)	25.89 ± 4.32	-	
Glucose (mg/dL)	92.17 ± 22.53	85.29 ± 9.97	
Urea (mg/dL)	98.30 ± 40.70	33.76 ± 9.64	<0.0001
SCr (mg/dL)	All patients 4.01 (6.22) KT 1.44 (0.91) Pre-dialysis 3.48 (0.74)	0.70 (0.08)	<0.0001
CKD-EPI (mL/min/1.73 m^2^)	All patients 13.12 (38.98) KT 51.94 (22.36) Pre-dialysis 17.97 (5.96)	98.47 (13.83)	<0.0001
Potassium (mEq/L)	4.64 ± 0.72	4.12 ± 0.29	0.0010
CRP (mg/L)	2.00 (4.10)	1.00 (0.80)	
Total cholesterol (mg/dL)	164.69 ± 40.68	179.29 ± 39.37	
Triglyceride (mg/dL)	118.89 ± 62.35	86.29 ± 32.69	
Proteinuria (mg/dL)	80.20 (522.35)	5.05 (93.80)	0.0003
Calcium (mg/dL)	9.14 ± 0.79	8.76 ± 0.32	0.0030
Phosphorus (mg/dL)	3.87 ± 1.20	3.33 ± 0.45	0.0018
Parathyroid hormone (PTH)	225.25 (238.6)	72.25 (34.05)	<0.0001
Klotho (pg/mL)	237.70 (570.24)	98.58 (325.76)	0.3123
Indoxyl sulfate (nmol/mL)	64.46 ± 73.25 35.37 (79.40)	10.01 ± 5.67 9.73 (6.85)	0.0005
IAA (µg/mL)	2.95 ± 13.06 0.00 (1.79)	0.10 ± 0.20 0.00 (0.00)	0.0397
pCS (µg/mL)	11.87 ± 13.57 7.96 (20.22)	1.62 ± 3.28 0.00 (0.00)	0.0006
RRT duration (days)	551.50 (1175.00)	-	

BMI: body mass index; SCr: seric creatinine; CRP: C-reactive protein; KT: kidney transplantation; CKD-EPI: Chronic Kidney Disease Epidemiology Collaboration; IAA: indole-3-acetic acid; pCS: p-Cresyl sulfate; and RRT: renal replacement treatment. KT (n = 24); pre-dialysis (n = 10).

**Table 3:** Klotho and uremic toxins comparative case–control analysis. IS: indoxyl sulfate; IAA: indole-3-acetic acid; and pCS: p-Cresyl sulfate.

Biomarker	Group	Mean ± SD	Median (IQR)	*p*-Value
Klotho (pg/mL)	Case (64)	343.08 ± 401.35	-	0.3123
	Control (16)	184.93 ± 228.28	-	
IS (nmol/mL)	Case (65)	64.46 ± 73.25	35.37 (79.40)	**<0.0001**
	Control (17)	10.01 ± 5.67	9.73 (6.85)	
IAA (µg/mL)	Case (65)	2.95 ± 13.06	0 (1.79)	**0.0397**
	Control (17)	0.10 ± 0.20	0 (0)	
pCS (µg/mL)	Case (65)	11.87 ± 13.57	7.96 (20.22)	**<0.0001**
	Control (17)	1.62 ± 3.28	0 (0)	

**Table 4:** Comparative analysis of biomarkers between peritoneal dialysis group and control group.

Biomarker	Group	Median (IQR)	*p*-Value
Klotho (pg/mL)	Case (20)	142.04 (306.94)	
	Control (16)	98.58 (325.76)	0.871
IS (nmol/mL)	Case (20)	68.48 (60.48)	
	Control (17)	9.73 (6.85)	**<0.0001**
IAA (µg/mL)	Case (20)	0.00 (0.68)	
	Control (17)	0.00 (0.00)	0.299
pCS (µg/mL)	Case (20)	2.83 (13.69)	
	Control (17)	0.00 (0.00)	**0.038**

**Table 5:** Comparative analysis of biomarkers between hemodialysis group and control group.

Biomarker	Group	Mean ± SD	Median (IQR)	*p*-Value
Klotho (pg/mL)	Case (12)	531.16 ± 442.73	-	
	Control (16)	184.93 ± 228.28	-	**0.032**
IS (nmol/mL)	Case (12)	169.76 ± 75.58	-	
	Control (17)	10.01 ± 5.67	-	**<0.0001**
IAA (µg/mL)	Case (12)	12.06 ± 29.45	1.99 (8.34)	
	Control (17)	0.10 ± 0.20	0.00 (0.00)	**0.003**
pCS (µg/mL)	Case (12)	20.10 ± 15.47	20.50 (20.45)	
	Control (17)	1.62 ± 3.28	0.00 (0.00)	**0.0006**

**Table 6:** Comparative analysis of biomarkers between pre-dialysis group and control group.

Biomarker	Group	Mean ± SD	Median (IQR)	*p*-Value
Klotho (pg/mL)	Case (10)	-	277.79 (682.12)	
	Control (16)	-	98.58 (325.76)	0.1116
IS (nmol/mL)	Case (10)	26.77 ± 15.87	-	
	Control (17)	10.01 ± 5.67	-	**0.0088**
IAA (µg/mL)	Case (10)	1.02 ± 1.11	0.58 (1.94)	
	Control (17)	0.10 ± 0.20	0.00 (0.00)	**0.0103**
pCS (µg/mL)	Case (10)	5.42 ± 7.56	1.33 (13.85)	
	Control (17)	1.62 ± 3.28	0.00 (0.00)	0.1271

**Table 7:** Comparative analysis of biomarkers between kidney transplant group and control group.

Biomarker	Group	Mean ± SD	Median (IQR)	*p*-Value
Klotho (pg/mL)	Case (23)	296.98 ± 343.29		0.2617
	Control (16)	184.93 ± 228.28		
IS (nmol/mL)	Case (23)	-	8.52 (14.90)	0.9891
	Control (16)	-	9.73 (6.85)	
IAA (µg/mL)	Case (23)	10.01 ± 5.67	0.00 (0.70)	0.3568
	Control (17)	0.67 ± 1.38	0.00 (0.00)	
pCS (µg/mL)	Case (23)	13.04 ± 13.37	11.03 (18.09)	**0.0002**
	Control (17)	1.62 ± 3.28	0.00 (0.00)	

**Table 8:** Spearman correlation of CKD-EPI and uremic toxins in patients in pre-dialysis and kidney transplantation.

Variable 1	Variable 2	r	*p* Value
CKD-EPI	Indoxyl sulfate (IS)	−0.780	<0.0001
	Indole-3-acetic acid (IAA)	−0.411	0.017
Indoxyl sulfate (IS)	Indole-3-acetic acid (IAA)	0.393	0.024

## Data Availability

The data presented in this study will be available upon request to the corresponding author.

## Abbreviations

BMI	Body mass index
CKD	Chronic kidney disease
CKD-EPI	Chronic kidney disease epidemiology collaboration
CRP	C-reactive protein
HD	Hemodialysis
IAA	Indole-3-acetic acid
IMT	Intima–media thickness
IQR	Interquartile range
IS	Indoxyl sulfate
KDIGO	Kidney Disease: Improving Global Outcomes
KT	Kidney transplantation
MCP-1	Monocyte chemoattractant protein-1
NO	Nitric oxide
pCS	p-Cresyl sulfate
PD	Peritoneal dialysis
ROS	Reactive oxygen species
RRT	Renal replacement therapy
SCr	Serum creatinine
SD	Standard deviation

## Appendix A

Bivariate analysis of the association between carotid IMT and categorical variables.

**Table A1:** Bivariate analysis involving carotid intima–media thickness (IMT).

Carotid IMT	Variable		Mean ± SD	Median (IQR)	*p*-Value
Left side IMT (mm)	Diabetes	No (54) Yes (14)	0.56 ± 0.14 0.66 ± 0.17		**0.0421**
	Smoker	No (47) Yes (21)	0.57 ± 0.16 0.61 ± 0.14		0.2001
	Hemodialysis	No (55) Yes (13)	0.55 ± 0.14 0.70 ± 0.15		**0.0011**
Right side IMT (mm)	Diabetes	No (54) Yes (14)	0.52 ± 0.13 0.62 ± 0.14		**0.0183**
	Smoker	No (47) Yes (21)	0.52 ± 0.13 0.60 ± 0.14		**0.0242**
	Atheromatous plaque	No (46) Yes (22)	0.51 ± 0.10 0.62 ± 0.18		**0.0147**
	Vascular calcification	No (37) Yes (28)	0.49 ± 0.10 0.63 ± 0.14		**0.0001**
	Osteoporosis	No (34) Yes (34)	0.50 ± 0.12 0.5 9 ± 0.15		0.0566
Mean IMT (mm)	Diabetes	No (54) Yes (14)	0.54 ± 0.11 0.64 ± 0.12		**0.006**
	Smoker	No (47) Yes (21)	0.54 ± 0.12 0.61 ± 0.12		**0.040**
	Atheromatous plaque	No (46) Yes (22)	0.54 ± 0.09 0.62 ± 0.15		**0.030**
	Vascular calcification	No (37) Yes (28)		0.53 (0.09) 0.63 (0.21)	**<0.0001**
	Osteoporosis	No (34) Yes (34)	0.53 ± 0.10 0.59 ± 0.13		**0.041**

Non-parametric comparison of uremic toxin levels by CKD clinical status.

**Table A2:** Uremic toxins according to clinical setting group. PD: peritoneal dialysis; HD: hemodialysis; and KT: kidney transplantation.

Biomarker	Group	Median (IQR)	*p*-Value
IAA µg/mL	PD (20)	0.00 (0.68)	0.0738
	HD (12)	2.00 (8.34)	
	Pre-dialysis (10)	0.58 (1.94)	
	KT (23)	0.00 (0.70)	
IS nmol/mL	PD (20)	68.48 (60.48)	**<0.0001** Pre-dialysis vs. HD (0.0014) PD vs. KT (0.0001) KT vs. HD (<0.0001)
	HD (12)	163.15 (90.78)	
	Pre-dialysis (10)	23.66 (21.29)	
	KT (23)	8.52 (14.90)	
pCS µg/mL	PD (20)	2.83 (13.69)	**0.0431** Pre-dialysis vs. HD: (0.0405)
	HD (12)	20.50 (20.45)	
	Pre-dialysis (10)	1.33 (13.85)	
	KT (23)	11.03 (18.09)	

ANOVA comparison of IMT levels by CKD clinical status.

**Table A3:** Carotid intima–media thickness according to clinical setting group. PD: peritoneal dialysis; HD: hemodialysis; and KT: kidney transplantation.

Biomarker IMT	Group	Mean ± SD	*p*-Value	
Left IMT (mm)	PD (24)	0.54 ± 0.10	0.016	*p* = 0.048
	HD (11)	0.68 ± 0.14		
	Pre-dialysis (10)	0.60 ± 0.21		
	KT (23)	0.57 ± 0.17		
Right IMT (mm)	PD (24)	0.50 ± 0.14		*p* = 0.15
	HD (11)	0.62 ± 0.14		
	Pre-dialysis (10)	0.53 ± 0.11		
	KT (23)	0.56 ± 0.15		
Mean IMT (mm)	PD (24)	0.52 ± 0.09	0.027	*p* = 0.042
	HD (11)	0.65 ± 0.12		
	Pre-dialysis (10)	0.57 ± 0.10		
	KT (23)	0.57 ± 0.14		
